# Simultaneous submucosal tunneling endoscopic septum division and submucosal tunneling endoscopic resection for epiphrenic diverticulum combined with an esophageal submucosal tumor

**DOI:** 10.1055/a-2647-4577

**Published:** 2025-07-29

**Authors:** Shao-Bin Luo, Zu-Qiang Liu, Li Wang, Quan-Lin Li, Ping-Hong Zhou

**Affiliations:** 1 92323 Endoscopy Center and Endoscopy Research Institute, Zhongshan Hospital Fudan University, Shanghai, China; 2 Shanghai Collaborative Innovation Center of Endoscopy, Shanghai, China; 3 729313 Endoscopy Center, Shanghai Geriatric Medical Center, Shanghai, China; 4 92323 Zhongshan Hospital Fudan University, Shanghai, China

A 43-year-old woman was admitted with a history of dysphagia for 2 years. Endoscopy showed an esophageal submucosal tumor and an epiphrenic diverticulum located above the esophagogastric junction (EGJ) ( Fig. 1 ). Submucosal tunneling endoscopic septum division (STESD) combined with submucosal tunneling endoscopic resection (STER) with was performed ( Video 1 ).

**Fig. 1:**
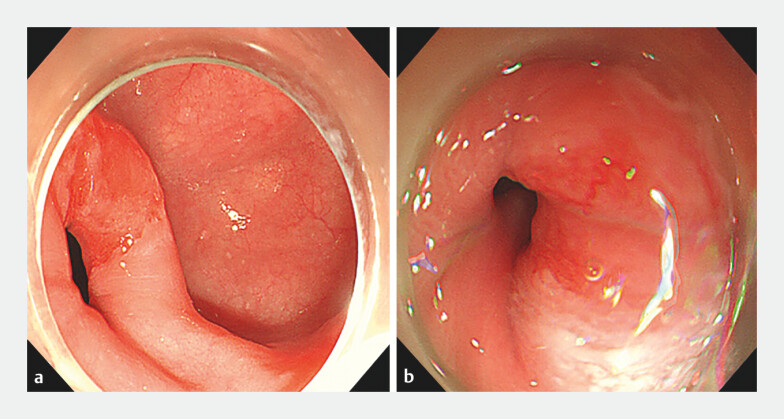
Endoscopic images showing: **a** an epiphrenic diverticulum; **b** a submucosal tumor in the cardia.

Simultaneous STESD and STER in one tunnel for an epiphrenic diverticulum combined with an esophageal submucosal tumor.Video 1

After the submucosal tunnel had been established, the annular muscle bundle and diverticular ridge were completely transected, and the whole layer of the esophageal muscle bundle was completely severed 2 cm above and below the EGJ ( Fig. 2 ). In addition, the esophageal muscularis propria above the cardia showed obvious thickening in the tunnel. A 4.0-cm submucosal tumor with unclear boundaries was found, and full-thickness resection was performed ( Fig. 3 ). The postoperative pathological diagnosis was leiomyoma. The patient was discharged on postoperative day 3 without complications. Follow-up endoscopy 1 year after the procedure confirmed the base of diverticulum was flattened ( Fig. 4 ), and the patient’s symptoms of dysphagia had disappeared.

**Fig. 2:**
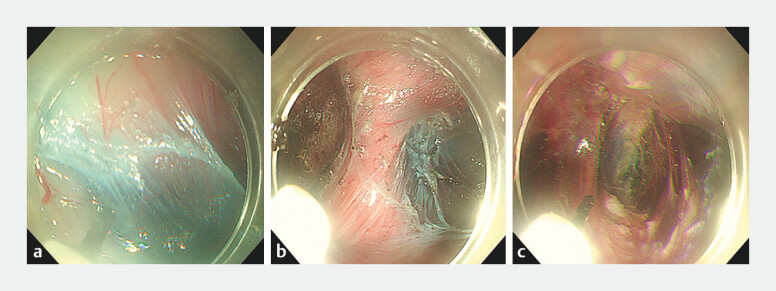
Endoscopic images showing **: a** the creation of a submucosal tunnel; **b, c** complete transection of the muscle of the diverticulum septum.

**Fig. 3:**
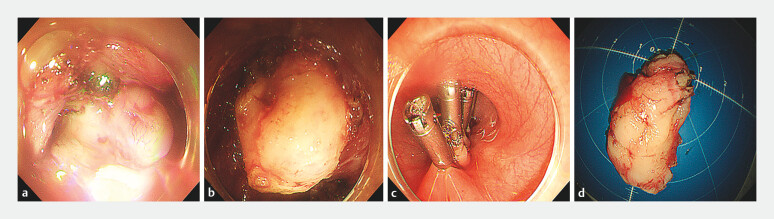
Endoscopic images showing: **a** a submucosal tumor with unclear boundaries **b** full-thickness resection being performed; **c** closure of the tunnel entrance with metal clips. **d** The resected specimen, which was 4 × 2 cm in size.

**Fig. 4:**
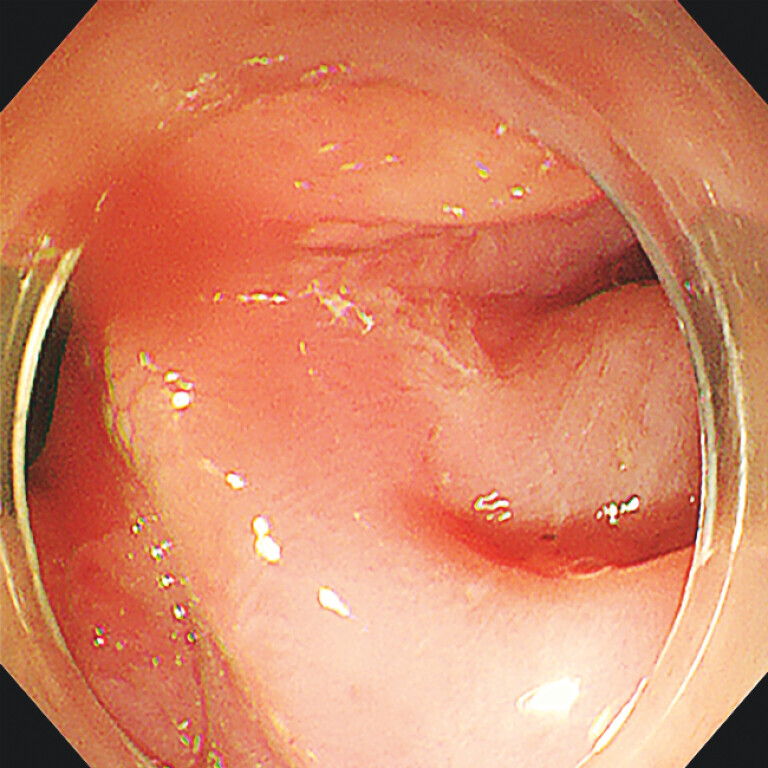
Endoscopic image at follow-up 1 year later showing the flattened base of diverticulum.

This case is the first report of simultaneous STESD and STER in one tunnel for an epiphrenic diverticulum with an esophageal submucosal tumor. Incision of the diverticular ridge and the spastic muscle layer within the same tunnel aimed avoid mucosal injury and esophageal perforation, which can greatly increase the difficulty of such procedures. The traditional treatment for an epiphrenic diverticulum with an esophageal submucosal tumor would be laparoscopic epiphrenic diverticulectomy, myotomy, and fundoplication, which is associated with high postoperative morbidity 1 . The advantage of STER is preservation of the overlying mucosal flap, which ensures the relative integrity of the esophageal wall, thereby reducing the risk of infection and pneumomediastinum.

This case suggests that the application of simultaneous STESD and STER in one tunnel may be a safe and effective technique for an epiphrenic diverticulum combined with an esophageal submucosal tumor.

Endoscopy_UCTN_Code_TTT_1AO_2AG_3AZ

## Notes

Endoscopy E-Videos https://eref.thieme.de/e-videos
*E-Videos* is an open access online section of the journal *Endoscopy* , reporting on interesting cases and new techniques in gastroenterological endoscopy. All papers include a high-quality video and are published with a Creative Commons CC-BY license. Endoscopy E-Videos qualify for HINARI discounts and waivers and eligibility is automatically checked during the submission process. We grant 100% waivers to articles whose corresponding authors are based in Group A countries and 50% waivers to those who are based in Group B countries as classified by Research4Life (see: https://www.research4life.org/access/eligibility/ ). This section has its own submission website at https://mc.manuscriptcentral.com/e-videos .
